# Behavioral stress induces regionally-distinct shifts of brain mineralocorticoid and glucocorticoid receptor levels

**DOI:** 10.3389/fnbeh.2014.00019

**Published:** 2014-01-29

**Authors:** Dorian Caudal, Thérèse M. Jay, Bill P. Godsil

**Affiliations:** Laboratoire de Physiopathologie des Maladies Psychiatriques, Centre de Psychiatrie et Neurosciences U894, INSERMParis, France; Faculté de Médecine Paris Descartes, Université Paris DescartesParis, France

**Keywords:** behavioral stress, mineralocorticoid receptors, glucocorticoid receptors, medial prefrontal cortex, hippocampus, amygdala

## Abstract

Mineralocorticoid and glucocorticoid receptors (MRs and GRs) mediate the impact of stress on brain function primarily by affecting gene transcription in the cell nucleus. *In vitro* studies using hippocampal neurons indicate that MRs and GRs translocate to the nucleus after binding to the stress hormone corticosterone, yet the *in vivo* temporal dynamics of MR and GR levels in other limbic regions critical for the stress response, however, are largely unknown. Rats underwent an elevated platform (EP) stress procedure and brain tissue was sampled from the amygdala (AMY), medial prefrontal cortex (mPFC), dorsal hippocampus and ventral hippocampus. By measuring MR and GR levels in the nuclear fraction from the tissue sampled, we observed striking shifts in the protein levels that varied by receptor, brain region and by the time after EP stress. These findings indicate that the subcellular trafficking of corticosteroid receptors display distinct temporal dynamics in different limbic regions after behavioral stress. These heterogeneous effects could underlie contrasting regional responses to stress within the brain, and they highlight the importance for systems-level analysis of stress responsivity.

## Introduction

The release of corticosteroids is a hallmark feature of the stress response and the actions of these hormones in the brain are pivotal in mediating both beneficial and detrimental neural adaptations to stress (de Kloet et al., 2005; McEwen, 2007). Corticosterone, the major stress hormone in rodents, exerts its effects through mineralocorticoid receptors (MRs) and glucocorticoid receptors (GRs), which operate as transcriptional regulators of gene expression (Reul and de Kloet, 1985; Funder, 1997; Herman et al., 2003). Without corticosterone, unbound MRs and GRs are thought to be mainly localized in the cytoplasm, but they can translocate to the nucleus after binding to the hormone ligand. With a much higher affinity for corticosterone compared to GRs, MRs are thought to be significantly occupied at basal levels (when MR ligands are present in low concentrations), whereas GRs become substantially activated only when corticosterone levels rise, such as after stress (de Kloet et al., 2005). These nuclear-localized MRs and GRs (nMR and nGR) initiate transcriptional processes that generate long-lasting stress effects which are typically manifested beginning 1 h later, while a different population of membrane-localized MRs and GRs receptors are believed to mediate rapid, non-genomic effects of stress hormones (Evanson et al., 2010; Pasricha et al., 2011; Tasker and Herman, 2011; Joels et al., 2012; Maggio and Segal, 2012).

MRs are heavily expressed in the hippocampus, but are also present in the amygdala (AMY) and medial prefrontal cortex (mPFC), while GRs are expressed more extensively throughout the brain (Reul and de Kloet, 1985). While the bulk of research on these brain receptors has focused on hypothalamic and hippocampal neurons, more recent work has shed light on their function in AMY and mPFC neurons (Kitchener et al., 2004; Karst et al., 2010; Yuen et al., 2011). Collectively, these studies suggest that MR- and GR- mediated mechanisms might vary by brain region. For example, hippocampal neurons showed rapid and reversible MR-dependent increases in excitability and glutamatergic transmission in response to corticosterone, whereas neurons of the basolateral AMY displayed long-lasting increases in excitability after one corticosterone treatment, but decreased excitability after a second corticosterone challenge in a manner involving both MRs and GRs (Karst et al., 2010). Contrasting effects of stress in different brain regions are also manifested by differential structural plasticity (Vyas et al., 2002), synaptic plasticity (Vouimba et al., 2004; Lee et al., 2011), BDNF release (Lakshminarasimhan and Chattarji, 2012), and glutamate receptor phosphorylation (Caudal et al., 2010). Such regional differences are an important issue because they imply that stress might alter entire networks in ways that cannot be understood without integrating stress effects across multiple brain regions.

Much of what is known about the subcellular dynamics of MRs and GRs come from *in vitro* studies involving hippocampal neurons maintained in culture (Nishi et al., 2001; Nishi and Kawata, 2006) and only a small number of reports have examined MRs or GRs in multiple brain regions in the same study (Nishi et al., 2001; Kitchener et al., 2004; Sarabdjitsingh et al., 2009). Consequently, we investigated how behavioral stress impacts the nuclear localization of MRs and GRs *in vivo* in four brain regions important for the stress response: the AMY, the mPFC, and the dorsal and ventral hippocampus (DH and VH).

## Materials and methods

### Animals

Experiments were performed with adult male Sprague-Dawley rats (300–400 g), housed 3–4 per cage. Rats were maintained in a temperature-controlled facility (22 ± 1°C) with a 12/12 h light/dark schedule (7:00 am/7:00 pm) and they had free access to food and water. Animals were kept at least 7 days after arrival from the supplier before being used in the experiment (*Charles River, L’Arbresle, France*). The stress protocol was performed during the beginning of the light phase (8:00 am–12:00 noon). All procedures were conducted in conformity with national (JO 887-848) and European (2010/63/EU) rules for animal experimentation. All efforts were made to minimize animal suffering and to reduce the number of animals used.

### Elevated platform (EP) stress and injection procedures

Elevated platform (EP) stress-treated rats were brought to an unfamiliar room where they were placed individually on an elevated and unsteady platform for 30 min. The platform dimensions were 20 × 21 cm and it was situated 100 cm above the ground. A bright fluorescent lamp (38 W, Goliath, JO-EL, Denmark; 1500 Lux) was positioned at the same height as the platform and its light beam was directed at the platform from 50 cm away. The lamp was included in the procedure because bright light is an ecologically relevant danger signal that evokes a low level of fear/stress in rats (File and Peet, 1980; Godsil and Fanselow, 2004). Moreover, bright light from fluorescent or incandescent light sources have been shown to elicit similar behavioral responses related to risk assessment (Godsil and Fanselow, 2004).

Immediately after the platform procedure rats were administered a sequence of two injections. The first injection was dimethyl sulfoxide (DMSO; 1 ml/kg i.p.) which was followed by an injection of the anesthetic sodium pentobarbital (60 mg/kg i.p.). Control rats (non-stressed rats) received the same sequence of injections as the EP rats while being briefly removed from their home cage. Afterwards, the body temperatures of all the rats were maintained with homeothermic warming blankets (37°C) until they were killed by decapitation 10 or 60 min later. The groups were *Control-10 min* (*n* = 5), *Control-60 min* (*n* = 6), *Stress-10 min* (*n* = 6), and *Stress-60 min* (*n* = 6).

The DMSO injections were included in this study to mimic the conditions of a previous report that demonstrated immediate post-stress GR blockade can reverse the stress-induced disruption of plasticity in the mPFC (Mailliet et al., 2008). Additionally, sodium pentobarbital was administered because we wished to retain continuity with our previous methodology for experiments that studied the impact of EP stress on *in vivo* electrophysiology in anesthetized rats (Rocher et al., 2004; Mailliet et al., 2008; Qi et al., 2009).

### Plasma corticosterone measurement

Trunk blood samples were collected just after decapitation. Samples were centrifuged at 1000 g for 15 min, and serum was stored at −20°C. Plasma corticosterone was assessed by immunoassay (*Corticosterone Immunoassay^®^, DSL, USA*).

### Tissue sample preparation

**Figure 1:**
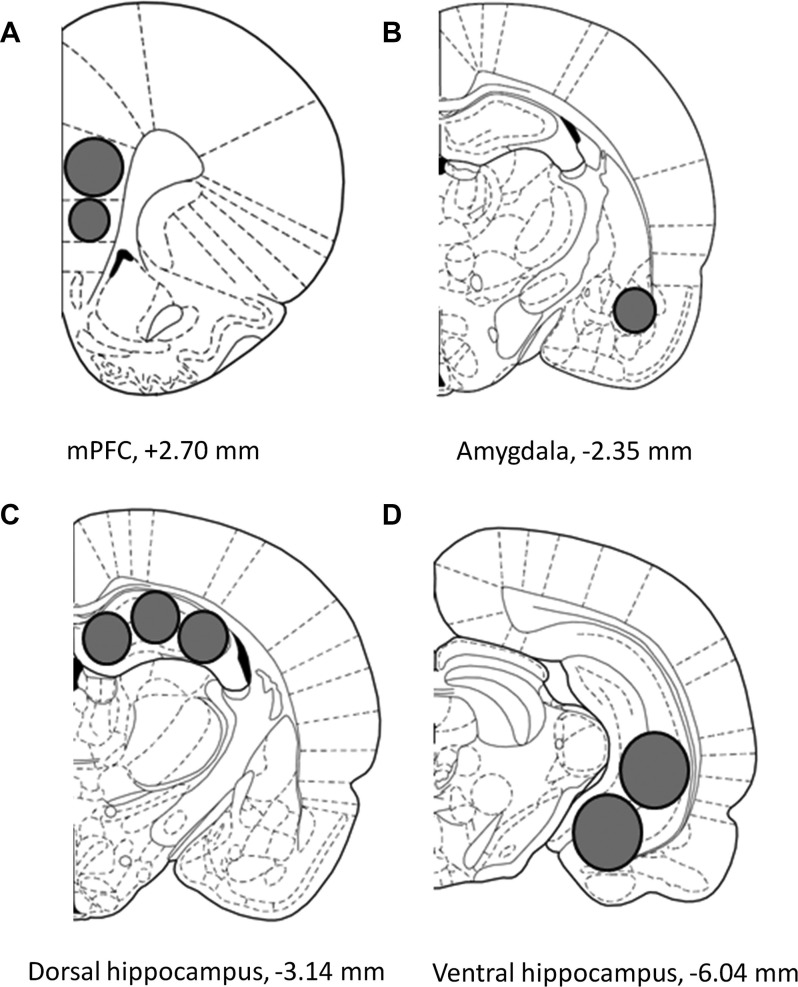
Schematic representation describing the location of the tissue sampled from the **(A)** medial prefrontal cortex (mPFC), **(B)** amygdala (AMY), **(C)** dorsal hippocampus and **(D)** ventral hippocampus. Images adapted from Paxinos and Watson (1998).

After decapitation, brains were snap-frozen in liquid nitrogen and stored at −80°C until processed. Using tissue punchers (0.50, 0.75 or 1.0 mm; *Harris Unicore, USA*), samples were extracted from 100 μm-thick sections prepared in a cryostat at −20°C. The mPFC (prelimbic and infralimbic) was sampled from 2.7 to 4.0 mm anterior of bregma. The AMY, DH, and VH were sampled from 2.0 to 3.5 mm, 3.0 to 4.6 mm, and 5.0 to 6.0 mm posterior of bregma, respectively, according to a rat brain atlas (Figure 1A–D; Paxinos and Watson, 1998).

### Isolation of nuclear fraction

Tissue was homogenized with a small Teflon-glass potter in ice-cold buffer containing 0.32 M sucrose, 1 mM HEPES, 1 mM MgCl_2_, 1 mM EDTA, 1 mM NaHCO_3_, and 0.1 mM phenylmethylsulfonyl fluoride (pH 7.4) and a protease inhibitor cocktail (*ProteoBlock, Fermentas, France*). The homogenized tissue was centrifuged at 1700 g for 15 min to separate a pellet (pel-1) enriched in nuclear components from the supernatant (sup-1). Pel-1 was washed three times in phosphate-buffered saline in order to avoid cytosolic contamination. The resulting supernatant (sup-1) was centrifuged at 13,000 g for 15 min. The obtained supernatant (sup-2) was a clarified fraction of cytosolic proteins and the pellet (pel-2), corresponding to a crude membrane fraction, was discarded. Protein concentrations were determined with a BCA kit using a Nanodrop ND-1000 spectrophotometer (*Thermo Scientific, France*).

### Western blotting

Samples (40 μg; 92 total) were separated twice on two different 4–15% running gels (MR and GR respectively; 26 wells, *Criterion^*TM*^ Precast Gel, Tris-HCl, Bio-Rad, France*) and transferred to a 0.2 µm polyvinylidene fluoride membrane (*Bio-Rad*). Membranes were incubated for 30 min at room temperature in blocking buffer (tris-buffered saline (TBS)-Tween 20 0.1%, bovine serum albumin 5%, NaN_3_ 0.02%). Immunoblotting was performed with either an anti-GR (1/200), an anti-MR (1/200) (M-20 and *H-300, Santa Cruz Biotechnology, Germany*), or an anti-actin antibody (1/5000) (*Millipore*, France). An anti-histone antibody (1/500) (MAB3422, Millipore) and an anti-cyclophilin A antibody (1/1000) (#2175, Cell Signaling Technology) were used to test the quality of the nuclear and cytoplasmic fractions, respectively. Membranes were washed three times with TBS-Tween 20 0.1% and incubated with secondary horseradish peroxidase (HRP) anti-rabbit antibody (dilution 1/1000) or HRP anti-mouse antibody (dilution 1/1000, only with the primary actin antibody) for 1 h at room temperature (*P.A.R.I.S, France*). At the end of the incubation, membranes were washed three times with TBS-Tween 20 and the immunoreactive bands were detected by chemiluminescence (Immun-Star^*TM*^ WesternC^*TM*^ kit,* Bio-Rad*). A series of primary, secondary antibody dilutions and exposure times were used to optimize the experimental conditions for the linear sensitivity range of the autoradiography films (*Santa Cruz Biotechnology, USA*). Films were scanned on the GS-800 Imaging Densitometer (*Bio-Rad*) and the density of each band was quantified using the Quantity One software (*Bio-Rad*).

### Data analysis

MR and GR protein quantities were calculated as the ratio to their actin loading control. Statistical analyses were performed with these ratio data. To assess the between-region differences of EP stress on protein quantities, nMR and nGR data (from control- and stress-rats) were entered into separate factorial ANOVAs for each receptor with factors for Stress (control and stress), Time (10 and 60 min), and Brain Region (AMY, mPFC, DH and VH). To further characterize the effect of EP stress on protein quantities, data from each brain region were entered into separate one-way ANOVAs for each protein followed by planned contrasts. For these analyses, Levene’s test for equality of variance was used. Data from groups with unequal variance were transformed with the log function before being submitted to a one-way ANOVA. Plasma corticosterone data were entered into a factorial ANOVA with factors for Stress and Time. A *p*-value of 0.05 was used as the criteria for statistical significance.

## Results

### The compartmentalization procedure isolated nuclear material

**Figure 2:**
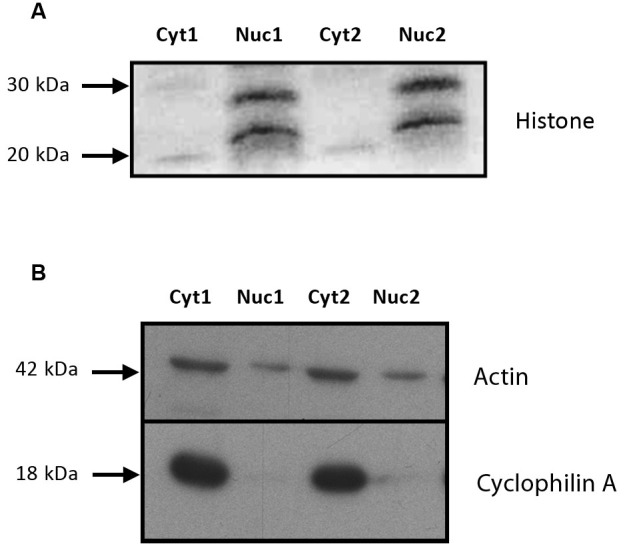
**The tissue isolation procedure produced high quality nuclear and cytosolic fractions. (A)** Immunoblot of histone proteins (20 and 28 kDa) in the nuclear and cytosolic fractions from the subcellular compartmentalization protocol used to isolate the nuclear material. An anti-histone antibody was used to assess the purity of the fraction. **(B)** Immunoblot of cyclophilin A and actin (18 and 42 kDa) in the nuclear and cytosolic fractions from the subcellular compartmentalization protocol used to isolate the nuclear material. An anti-cyclophilin A antibody was used to assess the purity of the fraction. This protocol was based on the technique used by Fumagalli et al. (2010). Cyt: cytosolic fraction, Nuc: nuclear fraction. 1 and 2 comprise for two independent samples (unstressed and stressed animals euthanized at 60 min).

Samples from both a nuclear and cytosolic fractions were tested for purity. As shown in Figure 2, a substantial amount of the nuclear marker histone was observed in the nuclear fraction, but not in the cytosolic fraction (Figure 2A). Conversely, the cytosolic marker cyclophilin A was absent from the nuclear fraction, but abundant in the cytosol (Figure 2B). Together, these results indicate that the compartmentalization procedure was successful at isolating nuclear material for the measurement of the MR and GR proteins.

### Nuclear mineralocorticoid receptor (MR) levels show distinct temporal changes in medial prefrontal cortex (mPFC) and hippocampus after exposure to elevated platform (EP) stress

**Figure 3:**
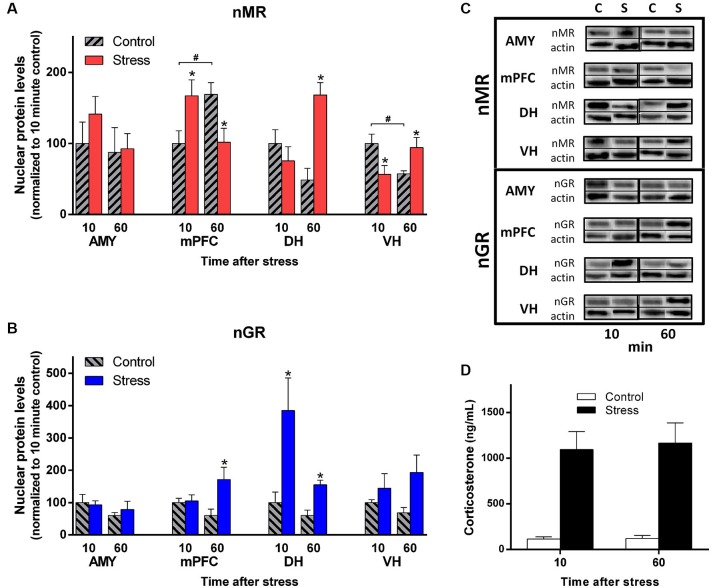
**EP stress causes regionally-distinct subcellular changes in MR and GR levels. (A)** MR levels detected in the nuclear fraction of the four brain regions. **(B)** GR levels detected in the nuclear fraction of the four brain regions. Tissue was sampled 10 and 60 min after EP stress. Data from are represented as the percentage of the 10 min control group for each region. * denotes a significant contrast compared to the control group of the same time point. ^#^ indicates a significant contrast between the control groups at 10 vs. 60 min. **(C)** Western blots of nMR and nGR levels in each brain region sampled 10 and 60 min after EP stress and in control conditions. For each timepoint, the column labels denote “Control” (“C”) and “Stress” (“S”). Upper immunoblots illustrate GR (95 kDa) or MR (102 kDa), and lower immunoblots illustrate actin (42 kDa). **(D)** Plasma corticosterone levels from trunk blood sampled 10 and 60 min after EP stress. Error bars denote standard error of the mean.

As seen in Figure 3A, C (top), exposure to EP stress caused changes in nMR levels that varied across the four different brain areas that were sampled. Indeed, compared to their controls, the AMY and mPFC tended to display decreasing nMR levels between 10 and 60 min after stress, whereas the DH and VH showed increases. This heterogeneity of nMR levels in response to behavioral stress was confirmed by a significant Stress × Time × Brain Region interaction (factorial ANOVA: *F*_(3,76)_ = 9.20, *p* < 0.0001). Thus, the temporal dynamics of nMR levels varied by brain region after EP stress.

To further characterize these stress effects, we analyzed the protein data from each region separately with one-way ANOVAs followed by planned contrasts. With this analysis, no group differences in nMR levels were detected in the AMY (*F*_(3,19)_ = 0.79, *p* = 0.51). In contrast, a complex pattern of group effects was observed in the mPFC (*F*_(3,19)_ = 3.98, *p* < 0.05). Exposure to stress was associated with increased mPFC nMR levels at 10 min, followed by decreased levels at 60 min compared to the corresponding control. Furthermore, control-treated rats showed elevated mPFC nMR at 60 min compared to10 min. Thus, in the mPFC both the stress and control treatments influenced MR levels in the nucleus.

Group differences were also detected in the hippocampus, but the pattern was different in each subregion. Stress elevated nMR at 60 min in the DH (*F*_(3,19)_ = 8.22, *p* < 0.002). In the VH the pattern was more complex (*F*_(3,19)_ = 3.98, *p* < 0.05). EP stress decreased nMR levels at 10 min, but increased them at 60 min. Additionally, the control treatment was associated with a decrease in VH nMR over time. Together, these results demonstrate nMR levels showed distinct patterns in response to the stress and control treatments across the different brain regions.

### Nuclear glucocorticoid receptor (GR) levels show distinct temporal changes in medial prefrontal cortex (mPFC) and hippocampus after exposure to elevated platform (EP) stress

Importantly, EP stress caused distinct changes in GR levels that were different from the MR pattern (Figure 3B, C (bottom)). Overall, nGR levels exhibited a significant interaction of Stress, Time, and Brain Region (factorial ANOVA: *F*_(3,76)_ = 3.05, *p* < 0.05) but the pattern was manifested in a different way than MR.

Considering each region separately with one-way ANOVAs, no group differences were detected in the AMY (*F*_(3,19)_ = 0.87, *p* = 0.48). In the PFC nGR levels were similar at 10 min, but elevated at 60 min (*F*_(3,19)_ = 3.52, *p* < 0.05). The group data from the DH displayed heterogeneous variance. Therefore, we employed a log transformation to the original values and submitted these transformed data to a one-way ANOVA. This analysis detected group differences (*F*_(3,19)_ = 6.05, *p* < 0.005) and planned contrasts revealed that nGR levels were elevated at both 10 and 60 min post stress. For the VH, nGR levels appeared slightly elevated at 60 min post stress, but this effect was not statistically significant (*F*_(3,19)_ = 1.97, *p* = 0.15). Notably, the control treatment did not influence nGR levels in any of the brain regions. Overall, these data demonstrate that the expression of MRs and GRs show distinct signatures across brain regions and time domains *in vivo* after behavioral stress.

### Exposure to elevated platform (EP) stress caused a high level of corticosterone in blood plasma relative to control conditions

Trunk blood was sampled from a subset of rats at the time of tissue harvesting. Ten and 60 min after injections, EP-treated rats showed robust elevation in plasma corticosterone levels while control rats had low levels. At the later timepoints EP-treated rats maintained high corticosterone levels, yet control rats also showed appreciable levels (Figure 3D; Main Effect of Stress *F*_(1,11)_ = 39.87, *p* < 0.0001). Thus, the stress procedure produced a sustained elevation in blood plasma corticosterone.

## Discussion

Regional differences in the brain’s response to stress imply that stress alters entire networks in ways that cannot be understood without characterizing the stress response at a systems level. Here we report that exposure to behavioral stress contributed to striking shifts in the levels of corticosteroid receptors that varied by brain region and by time after stress. Generally, EP stress transiently elevated nMR levels in mPFC followed by a decrease, whereas hippocampal tissue displayed control levels, or initial decreases in nMR levels, followed by increases at a delayed timepoint (60 min) and the AMY did not show any elevation. In contrast, nGR proteins were elevated in the DH after EP stress (at 10 and 60 min), while enhanced nGR levels were observed in the mPFC at longer latencies (60 min) and no elevation was found in the AMY. These findings provide evidence showing that the subcellular dynamics of MRs and GRs in response to a sustained behavioral stressor are distinct in different limbic regions, and they are consistent with the general pattern that MRs and GRs can have differential involvement shortly after stress compared to a long delay (Chaouloff and Groc, 2011; Joels et al., 2012).

The predominant account describing the subcellular trafficking of corticosteroid receptors holds that, in the absence of their ligand, MRs and GRs are mainly present in the cytoplasm and they rapidly translocate to the nucleus after the initiation of stress (Nishi and Kawata, 2007; Joels et al., 2012). This view is founded on a variety of data, including measurement of fluorescent-tagged corticosteroid receptor proteins in living cell cultures after exposure to corticosterone (Fejes-Toth et al., 1998; Nishi et al., 2001). Based on this assumption one might expect to observe global, rapid increases in nuclear MRs and GRs over time, yet some of our results seem to contradict this prediction. For example, we observed elevations in MRs in the DH, and in GRs in the mPFC, 60 min after stress, but not sooner. Moreover, we also observed cases where corticosteroid receptors were decreased in the nucleus after stress.

In considering these facts, it is important to be mindful that the first protein samples were collected 10 min after the end of our 30 min stress protocol. Thus, our procedure tracks the changes in corticosteroid receptors *after the cessation* of a behavioral stressor, and it does not measure the initial translocation events that occur shortly after the initiation of stress. Nevertheless, our results are generally consistent with one study that measured nGR levels immediately after 30 min of behavioral stress (Kitchener et al., 2004). By this time point, a substantial amount of nGR was detected and nGR levels decreased at later timepoints in both the hippocampus and PFC. Taken together with our results, it appears that following the initial translocation events to the nucleus at the initiation of stress, corticosteroid receptors return to the cytoplasm, or are degraded, with varying temporal dynamics depending on the brain region. To our knowledge, this is the first study documenting nuclear-localized distributions of MRs and GRs across several brain regions.

Another consideration is that the localization of MRs in ligand-free conditions remains controversial (Fejes-Toth et al., 1998; Nishi et al., 2011). Some evidence suggests that the receptors are distributed in both the cytoplasm and nucleus (Fejes-Toth et al., 1998), just as translocation processes can vary by cell type and by corticosterone concentration (Nishi et al., 2001; Sarabdjitsingh et al., 2009). Also, our sampling method does not discriminate between cell types within a region (such as pyramidal neurons vs. interneurons, astrocytes, or oligodendrocytes). To our knowledge the relative contributions of different cell types within a brain region to stress responsivity are poorly characterized. But it is noteworthy that glial cells express both MRs and GRs (Bohn et al., 1991), especially because these brain cells appear to contribute to stress-induced brain pathology (Banasr et al., 2010). Our results provide initial evidence that the stress-evoked dynamics of MR and GR levels appear to be distinct in different brain areas, but clearly more research is needed to clarify the precise nature of these differences.

In our experiments, we detected high levels of plasma corticosterone at both 10 and 60 min after the EP stress procedure. Using a different version of an EP stress procedure, however, Degroot et al. (2004) reported that corticosterone recovered to control levels by 60 min post stress. This disparity can likely be accounted for by important differences in the experimental protocols. In the Degroot experiment, rats were extensively handled prior to the EP stress exposure, and the rats were familiar with the experimental room prior to testing. In our experiment, the rats were minimally handled prior to the testing day. Also, the stress-treated rats were brought to a novel room for the EP procedure, which had a 30 min duration and involved bright illumination. These procedural differences are important factors because handling, environmental novelty and bright illumination are known to increase the rat’s corticosterone response (Friedman et al., 1967; Brown and Martin, 1974; File and Peet, 1980). Thus, it is likely that our version of the EP stress procedure was more potent at evoking corticosterone release, which could account for the sustained elevation in plasma corticosterone that persisted at 60 min after the end of stress. Similar sustained corticosterone levels have also been observed with other acute stressors, including water immersion and immobilization (De Boer et al., 1990; Muñoz-Abellán et al., 2008).

It is also relevant to consider that our experiment was conducted with rats that were under the influence of pentobarbital anesthesia, which could influence the translocation patterns reported here, as well as the sustained corticosterone levels. Using the same procedure, we have observed a moderate elevation in plasma corticosterone in control-treated animals at 30 min post stress (unpublished). Thus, although not as intense as the EP stress treatment, the control treatment does evoke a stress response in the rats. Part of this response likely results from the i.p. injections (Barrett and Stockham, 1963), but it is also possible that the sodium pentobarbital anesthesia affects the hormone levels. Previous studies have suggested that corticosterone levels can rise in the presence of pentobarbital, however (Oliver and Troop, 1963), so the stress response is still active. Future studies could clarify the influence of this anesthetic on nMR and nGR levels in relation to stress.

It is also notable, and perhaps important, that the time course for the rise and fall of corticosterone levels can vary by brain region after stress (Dorey et al., 2012). Using a double microdialysis approach, it was demonstrated recently that the time course of corticosterone concentration changes after footshock stress is different in the DH compared to the VH. Specifically, corticosterone levels rise more rapidly in the DH (15–60 min after stress) compared to the VH (90–105 min after stress), and they return to baseline levels more quickly in the DH (105 min) compared to the VH (120 min). These time course differences could contribute to the regional differences we observed in these two structures. It would be interesting to measure corticosteroid receptor levels at later time points to determine whether the VH might show changes that are comparable to the DH, but which are manifested only at a further post-stress delay.

In recent years, there has been growing interest in membrane-localized MRs and GRs, which are thought to mediate rapid, non-genomic effects of stress (Groeneweg et al., 2011; Tasker and Herman, 2011; Joels et al., 2012). It is has been proposed that MRs and GRs might shuttle to the membrane region of neurons in response to high stress (Karst et al., 2005), where they could mediate rapid effects of stress hormones (Evanson et al., 2010; Tasker and Herman, 2011; Joels et al., 2012), perhaps in a manner similar to how estrogen receptors are inserted in the membrane (Dominguez and Micevych, 2010). Owing to the delay between the initiation of stress and its subsequent genomic effects, the data we present from 10 min post stress could possibly correspond to this non-genomic window, but additional control measures would be necessary to prove this point. Also, given the high corticosterone levels evoked in our procedure a subpopulation of corticosteroid receptors might possibly shuttle to the membrane along with the translocations destined for the nucleus. Future studies could address these issues, by studying protein levels with a procedure that isolates the membrane fraction of the cells.

MRs and GRs play a pivotal role in mediating the effects of stress on the brain. It has been known for decades that these receptors have differential expression patterns. Here we call attention to apparent regionally-distinct changes in corticosteroid receptor nuclear levels in response to behavioral stress. The functional significance of these differences is presently unknown, but they might support the aforementioned regional differences in structural plasticity, synaptic plasticity, and synaptic transmission (Vyas et al., 2002; Vouimba et al., 2004; Karst et al., 2010; Lee et al., 2011), which might be key in determining susceptibility to stressors (de Kloet et al., 2005). The changes we observed might also contribute to regional differences in the expression of membrane-associated MRs and GRs (Karst et al., 2005; Groeneweg et al., 2011; Joels et al., 2012), which are important for rapid adaptations to stress. Our finding, that acute stress caused a delayed increase in nGR after stress could underlie how stress disrupts plasticity in the mPFC (Rocher et al., 2004). This hypothesis is consistent with the observation that post-stress GR blockade reverses the stress induced disruption of such plasticity (Mailliet et al., 2008). By extension, regional differences in hippocampal and prefrontal stress mechanisms might be relevant for understanding the deficits in cognitive processing and emotional regulation that appear in multiple psychiatric disorders (Pêgo et al., 2010; Godsil et al., 2013). Indeed, given the impact of stress on the expression of psychiatric symptoms, elucidating these regional and subcellular differences should yield novel and clinically relevant insight into the stress response.

## Author contributions

All the authors contributed to the design, acquisition, analysis and interpretation of the data for this work. They all contributed to writing and have approved the final manuscript and agree to be accountable for all aspects of the work ensuring that questions related to the accuracy or integrity of any part of the work are appropriately investigated and resolved.

## Conflict of interest statement

The authors declare that the research was conducted in the absence of any commercial or financial relationships that could be construed as a potential conflict of interest.
